# Effectiveness and safety of intra-articular hyaluronic acid SEMICAL GEL-B CROSS therapy in knee osteoarthritis (SEM-ART1): Study protocol for a randomized, placebo controlled, double-blind, cross-over clinical trial

**DOI:** 10.1371/journal.pone.0353120

**Published:** 2026-07-09

**Authors:** Meral Bilgilisoy Filiz, Hanife Hale Hekim, Ahmet Bal, Aslıhan Kara

**Affiliations:** 1 Department of Physical Medicine and Rehabilitation, Health Sciences University, Antalya Training and Research Hospital, Antalya, Turkey; 2 Biologist, Medical Device Regulation (MDR) Compliance Specialist, Antalya, Turkey; Ondokuz Mayıs Üniversitesi: Ondokuz Mayis Universitesi, TÜRKIYE

## Abstract

**Objective:**

This study aims to evaluate the effectiveness and safety of a single intra-articular injection of high-dose cross-linked hyaluronic acid on pain, physical function, muscle strength, and quality of life in patients with knee osteoarthritis.

**Methods:**

This randomized, double-blind, placebo-controlled, cross-over clinical trial includes 102 adults with symptomatic knee osteoarthritis (Kellgren–Lawrence grade II–III). Participants will be allocated using stratified randomization based on radiographic severity and laterality. Patients will receive either intra-articular hyaluronic acid (Semical Gel-B Cross, 90 mg/3 mL) or isotonic saline (3 mL of 0.9% NaCl), followed by cross-over at the 3-month evaluation. Outcomes will be assessed at baseline and at 3, 6, 9, and 12 months.

The primary efficacy endpoint is the between-group difference in WOMAC pain score at the 3-month assessment prior to cross-over. Secondary outcomes include VAS pain, WOMAC physical function and stiffness scores, isometric quadriceps and hamstring strength, quality of life (SF-36), the 6-minute walk test, the 5-times sit-to-stand test, analgesic use, and adverse events. Treatment response will additionally be categorized as “absolute responders” (≥50% and ≥20-point improvement in VAS) and “responders” (≥20% and ≥10-point improvement). Sample size was calculated using G*Power (effect size 0.5, 80% power), yielding 102 participants. Statistical analyses will include both intention-to-treat and per-protocol approaches.

**Expected results and conclusions:**

This study will provide 3-month efficacy data and long-term (3–12 months) safety outcomes for high-dose intra-articular hyaluronic acid compared with placebo. Findings are expected to inform clinical decision-making regarding symptom relief, functional improvement, and the safety profile of cross-linked HA formulations in knee osteoarthritis.

**Trial registration:**

ClinicalTrials.gov: NCT06141018

## Introduction

Knee osteoarthritis (KOA) is a leading cause of pain and disability worldwide, and its prevalence is increasing due to population aging and obesity. According to a recent Global Burden of Disease analysis, 595 million people were living with osteoarthritis in 2020, accounting for 7.6% of the global population. KOA was the most frequently affected site, and the number of cases is projected to increase substantially by 2050 [1].

Core non-surgical management emphasizes patient education, exercise, weight management, and analgesia. However, the optimal use of intra-articular therapies is still debated among the various guidelines. The 2019 Osteoarthritis Research Society International (OARSI) guideline offers personalized, conditional recommendations for intra-articular therapies in KOA, whereas the 2019–2020 American Collage of Rheumatology (ACR)-Arthritis Foundation guideline cautions against the use of intra-articular hyaluronic acid (IA-HA) for KOA [2,3].

Hyaluronic acid (HA) is an important component of synovial fluid and joint cartilage, and due to its high viscosity, it contributes to joint lubrication and viscoelastic properties [4,5]. In patients with KOA, both the concentration and molecular weight of HA in synovial fluid are significantly reduced compared to healthy joints, leading to impaired joint biomechanics and increased friction [4,5]. IA-HA visco-supplementation aims to restore synovial fluid rheology and may exert analgesic and disease-modifying effects through viscoelastic lubrication, anti-inflammatory signaling (e.g., NF-kB downregulation), nociceptive modulation, and chondro-protection [6]. While some investigators attribute clinical benefits primarily to visco-supplementation and biomechanical restoration, others proposed additional anti-inflammatory, chondro-protective, and analgesic mechanisms, and some remain skeptical that visco-supplementation alone accounts for all observed improvements [7].

Evidence regarding symptomatic benefit is mixed. A 2022 systematic review/meta-analysis concluded that visco-supplementation provides a small reduction in pain compared to placebo, which may be below the threshold of minimal clinical important differences, and carries a higher risk of serious adverse effects compared to placebo [8]. Conversely, other contemporary umbrella/narrative reviews and comparative studies highlight the heterogeneity in products, populations, and trial methods, suggesting benefits in selected patients and/or with specific molecular weight preparations or multiple injection regimens [9,10].

Safety is generally acceptable, with mostly transient local reactions; rare but noteworthy events include pseudoseptic (“acute serious localized”) reactions that require careful differentiation from septic arthritis. Meta-analyses are not in agreement regarding the magnitude of the increased risk of adverse events; this reinforces the need for well-designed, blinded studies with systematic safety monitoring [8,11,12].

Methodologically, intra-articular saline comparators are not biologically inert; they can provide clinically meaningful pain relief and thus reduce the observed treatment-placebo contrast. This rather large “placebo”/contextual effect in IA injection trials complicates parallel comparisons between patients [13]. Given the heterogeneity of prior studies and the strong placebo effects associated with intra-articular interventions, randomized, double-blind, cross-over clinical trials provide an efficient and methodologically robust design to compare treatments within the same individuals, thereby minimizing inter-patient variability.

Therefore, the present study aims to evaluate the effectiveness and safety of an IA-HA agent SEMICAL Gel-B Cross 90 mg/ 3 ml (Semikal Teknoloji, Antalya, Turkey) in patients with symptomatic KOA using a randomized, placebo-controlled, double-blind, cross-over design, with comprehensive outcome assessments for pain, function, quality of life, and safety over a 12-month follow-up period.

## Materials and methods

This study protocol is reported in accordance with the Standard Protocol Items: Recommendations for Intervention Trials (SPIRIT) 2025 criteria [14] and designed as a randomized, placebo-controlled, double-blind, cross-over clinical trial. The protocol has been approved by the clinical trials ethics committee of Akdeniz University (KAD-FR-42/12-06-2024) and Turkish Medicines and Medical Devices Agency (E-68869993-000-1538194/2024-025). All participants has provided written informed consent prior to enrollment. The trial is being conducted in accordance with the principles of the Declaration of Helsinki and Good Clinical Practice guidelines. Participant recruitment has started in March 2025, and completed in December 2025. Data collection is expected to be completed in December 2026. The original protocol is provided as Supporting Information (S1 File and S2 File), and the completed SPIRIT 2025 checklist is provided as S3 File.

### Eligibility criteria

To be enrolled in this trial, the following eligibility criteria, assessed at screening, will be met:

Inclusion criteria:

Patients aged 18 years and older with a clinical diagnosis of KOA according to the ACR criteria, with idiopathic/primary knee osteoarthritis classified as Stage II and III according to the Kellgren & Lawrence (KL) grading system, will be evaluated for inclusion to the study, along with the following criteria:

- Pre-treatment pain rating of 4 points or higher on the VAS
- Indicated for intra-articular hyaluronic acid injection
- Previously received conservative treatment for knee osteoarthritis and did not achieve an adequate response (conservative treatment: exercise, non-steroidal anti-inflammatory drugs, physical therapy)
- Body mass index (BMI) between 20 and 40 kg/m²
- Able to provide written informed consent
- Not pregnant or breastfeeding
- At least two years postmenopausal or surgically sterile or fertile but willing to use acceptable methods of contraception
- Patients who agree to complete the washout period for non-steroidal anti-inflammatory drugs prior to the study procedures and administration of the study treatment (this means not taking any NSAIDs or other painkillers for at least 48 hours prior to visits where responses to the study treatment will be assessed).
- Able to walk independently without the aid of walkers, canes, crutches, or other assistive devices,
- With sufficient mental function to understand and correctly answer the questionnaires and scales used to evaluate treatment response (Mini Mental Test score above 24)
- Those for whom the DN-4 questionnaire questions can confirm that the pain is not neuropathic in origin,
- For those with bilateral KOA: provided that the VAS pain scores for both knees are similar (difference < 20).

Exclusion criteria:

- Pregnant women, breastfeeding women, and women planning to become pregnant within one year
- Patients diagnosed with autoimmune or inflammatory rheumatic diseases such as rheumatoid arthritis, gout, pseudogout, psoriasis, SLE, fibromyalgia
- Patients with active inflammation or infection
- Patients using anticoagulant therapy
- Patients with known allergies to hyaluronic acid and other excipients
- Other joint diseases that may interfere with the observation of treatment efficacy
- Other intra-articular injection treatments such as steroids, PRP, or stem cell injections within the previous 6 months prior to inclusion in the study
- Use of aspirin, acetaminophen, or other non-steroidal anti-inflammatory drugs within 48 hours prior to the study treatment, or use of other opioid, cannabinoid, and pyrazolone derivative analgesics
- Intra-articular hyaluronic acid injection within the 6 months prior to inclusion in the study
- Open surgical intervention on either knee within the last year
- Advanced osteoarthritis (KL Stage III-IV) in the hip joint
- Other treatments or practices (alternative medicine, nutritional supplements, etc.) that could produce results conflicting with the study treatment and procedures
- Peripheral neuropathy, vascular insufficiency, hemiparesis, systemic bleeding disorders
- Skin diseases or infections in the knee area where the injection will be administered
- Having osteoarthritis secondary to systemic metabolic diseases, or other painful musculoskeletal diseases
-  > 20 ml of synovial fluid during aspiration during the injection
- Those with varus or valgus deformity greater than 10 degrees or joint motion restriction greater than 10 degrees
- Patients receiving corticosteroid or other immunosuppressive drug therapy at a dose higher than 5 mg prednisolone daily
- Patients whose cognitive functions are insufficient for the assessment of outcome measures (patients with a mental score lower than 24 on the Mini Mental State Examination)
- Patients with planned permanent relocation outside the city or country, major surgery, incarceration, military service, quarantine, etc., for the 12 months during which study follow-ups will take place

### Randomization and interventions

Randomization and treatment allocation were generated before participant enrollment using a computer-generated stratified allocation list prepared in Microsoft Excel. Stratification was based on Kellgren–Lawrence grade (II vs III) and disease laterality (unilateral vs bilateral), resulting in four strata. Allocation codes were stored in a password-protected master list accessible only to Investigator-1, the unblinded investigator responsible for treatment preparation and administration. Treatment assignments were placed in sequentially numbered opaque sealed envelopes. Following confirmation of eligibility and informed consent, the participant’s study number was matched with the corresponding treatment code, and the envelope was opened by Investigator-1 immediately prior to treatment preparation. Outcome assessors and participants remained blinded throughout the study. The participants were assigned to one of the following groups:

- **Group A**: intra-articular hyaluronic acid (Semical Gel-B Cross, 90 mg/3 ml, cross-linked formulation).
- **Group B**: intra-articular isotonic saline (0.9%, 3 ml).

At the 3-month assessment, following the initial response evaluation, patients will be crossed over to the alternative treatment arm: those who initially received hyaluronic acid will be given isotonic saline, and vice versa. This procedure will also be performed by the unblinded research assistant, with careful attention paid to maintaining blinding. As a result, all patients will receive both treatments during the study period, ensuring that each participant has access to hyaluronic acid treatment in either the first or second injection.

To minimize the risk of unblinding, outcome assessors were fully separated from treatment preparation and injection procedures. Participants were not allowed to observe syringe labeling, preparation, or handling. Allocation information remained accessible only to Investigator-1 and the manufacturer. Outcome assessors will not be present during injection preparation or administration and will not have access to the allocation list, envelopes, syringe serial numbers, or treatment-preparation records. A single-injection regimen of 90 mg of cross-linked hyaluronic acid was selected for this study for two primary reasons. First, the use of a higher-dose formulation allows the evaluation of the long-term effectiveness and duration of therapeutic benefit over a 12-month follow-up period, providing valuable information on sustained clinical outcomes after a single intra-articular administration.

Second, the selected formulation is a highly cross-linked hyaluronic acid product. Cross-linking agents such as BDDE (1,4-butanediol diglycidyl ether) are commonly used in HA-based medical products, and residual cross-linker content represents an important quality-control and biocompatibility parameter. Therefore, this formulation was considered appropriate for systematic monitoring of local tolerability, hypersensitivity-type reactions, and other potential adverse events associated with highly cross-linked HA products.

For participants with bilateral knee osteoarthritis, both knees received the allocated intervention. However, efficacy analyses were performed using a pre-defined index knee. The index knee was defined as the more symptomatic knee at baseline. If symptom severity was identical, the knee with the higher radiographic grade was selected; if radiographic grades were also identical, the right knee was designated as the index knee. All WOMAC-based efficacy analyses and knee-specific outcome assessments were referenced to the index knee.

### Study procedure and time points

All study related activities will be performed at the outpatient clinics of Physical Medicine and Rehabilitation department of a tertiary level hospital.

Participants were asked to discontinue nonsteroidal anti-inflammatory drugs and other analgesics for knee pain (except paracetamol) from 2 days before baseline assessment through 12-month follow-up. Paracetamol (up to 3000 mg/day) will be allowed to use as rescue pain relief during the study.

Prior to randomization, screening and baseline assessments were performed. Patients underwent clinical assessment and laboratory testing, including a complete blood count, erythrocyte sedimentation rate, C-reactive protein, rheumatoid factor, and a urine test for premenopausal women to rule out pregnancy. The classification regarding the degree of osteoarthritis was performed by analyzing the radiograph of the affected knee/knees according to the method of KL.

**Fig 1:**
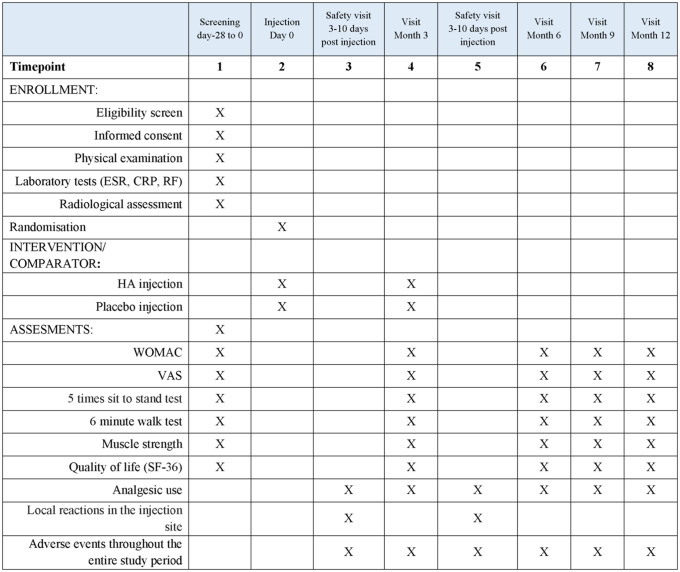
Participant timeline: Schedule of enrollment, interventions, and assessments. Abbreviations: CRP: C-reactive protein; ESR: eritrosit sedimentation rate; HA: hyaluronic acid; RF:rheumatoid factor; SF-36: short form-36; VAS: visual analogue scale; WOMAC: Western Ontario and McMaster Universities Osteoarthritis Index.

All patients will undergo an intra-articular injection at baseline. A safety assessment visit will be conducted between day 3 and day 10 to monitor for early adverse events.The first efficacy evaluation will take place at 3 months post-injection, which will serve as the cross-over point. At this visit, patients initially randomized to HA will receive placebo, and those initially randomized to placebo will receive HA. 3–10 days after the second injection, another safety visit will be performed. Further follow-up assessments will occur at 6, 9, and 12 months to evaluate the persistence of treatment effects. Study timeline is outlined in Fig 1.

#### Injection procedure.

Gel-B Cross 90 mg/ 3 ml (Semikal Teknoloji, Antalya, Turkey) is a cross-linked viscoelastic solution containing high molecular weight HA (2500–3000 kDa) obtained by bio-fermentation. The product is supplied as sterile, in a single-use pre-filled syringe of 3-mL containing 90 mg/mL HA. The comparator will consist of 3 mL of isotonic saline, produced by the same manufacturer as the active product. It will be supplied in identical prefilled syringes, containing 3 mL of 0.9% sodium chloride solution, and will be identical in appearance, volume, and packaging to the hyaluronic acid injection in order to maintain blinding. Each pre-filled syringe has a unique serial number. Only the manufacturer and the unblinded researcher know which numbers contain hyaluronic acid or placebo. Injections are also administered by the investigator who knows the contents of the serial numbers.

The intra-articular knee injections will be guided by ultrasound. All injections will be performed with the patients positioned supine on the examination table, and the lower extremities maintained in full extension throughout the procedure as recommended by EUROpean Visco-supplementation Consensus group (EUROVISCO) [15]. A 21-gauge, 50 mm needle will be inserted from the upper external area of the knee patella (suprapatellar) under sterile conditions. If present, aspiration will be performed using a separate syringe, and the volume of the aspirate will be noted. If the synovial fluid appearance is abnormal (i.e., cloudy, opaque and/or coloured), or more than 20 cc, treatment injection will not proceed. Following the injection, 5 passive knee flexion/extension movements will be performed, and participants will be remained rested for 10 minutes.

### Outcome measures

The primary efficacy endpoint of the study is the between-group difference in WOMAC pain score at the 3-month assessment, before cross-over, using the index knee and adjusted for baseline WOMAC pain score and stratification factors. Secondary outcome measures include pain scores measured by visual analogue scale (VAS) during rest and activity; WOMAC functional score; muscle strength assessed by myometer; functional measures such as the 5 times sit-to-stand test and 6-minute walk test; quality of life assessed by SF-36, Outcome Measures in Rheumatology Clinical Trials (OMERACT) – OARSI responder index, analgesic usage and rate of adverse events.

#### Western Ontario and McMaster Universities osteoarthritis index.

The WOMAC is a validated, patient-reported outcome measure commonly used to assess pain, stiffness, and physical function in individuals with lower extremity osteoarthritis [16]. It is the most widely used condition-specific instrument for the assessment of hip or KOA and is recommended by OMERACT [17]. WOMAC is considered a sensitive measure in clinical trials evaluating pharmacological, surgical, biological, and physical interventions and is recommended as an important efficacy endpoint by regulatory authorities such as the US Food and Drug Administration (FDA) and the European Medicines Agency (EMA) [18].

The WOMAC consists of 24 items grouped into three subscales: pain (5 items), stiffness (2 items), and physical function (17 items). Each item is scored on a 5-point Likert scale (1 = none, 2 = mild, 3 = moderate, 4 = severe, 5 = extreme). The possible score ranges are 0–20 for pain, 0–8 for stiffness, and 0–68 for physical function, with higher scores indicating greater pain, stiffness, and functional limitation. Patients are asked to evaluate their symptoms based on their experience during the previous 48 hours.

WOMAC has demonstrated excellent reliability, validity, and responsiveness in various clinical settings and has also been translated and validated in Turkish populations [19]. Its established psychometric properties make it one of the highest-performing outcome measures for assessing symptom severity and treatment effectiveness in KOA [20].

Overall average knee pain intensity over the last 48 hours will be assessed by a VAS with terminal descriptors of ‘no pain’ (recorded as 0) and ‘maximal pain’ (recorded as 10) for both rest and activity.

#### Muscle strength.

Strength of the quadriceps and hamstring muscles will be measured via a hand-held dynamometer (The Kinvent K-Push Dynamometer, Kinvent Physio, Montpellier, France) [21]. Each participant will be positioned on the examination table with their legs hanging down without touching the ground, knees and hips flexed at 90°. The torso will be maintained in an upright position,with arms extended and holding onto the examination table for support and ensuring stable body position. The maximum voluntary quadriceps muscle strength will be measured by placing the hand-held dynamometer on the front of the distal tibia and the maximum voluntary isometric hamstring muscle strength on the back of the lower leg. Participants will be instructed to flex or extend their knees “as hard as possible” toward the dynamometer and continue to apply force for five seconds. The maximum force they can generate during the experiment will be recorded [21].

#### 36-ıtem short form survey.

The SF-36 includes eight multi-item subscales, each containing two to ten items plus a single item, to assess health status. The scales range from 0 (maximum symptoms/maximum limitations/poor health) to 100 (no symptoms/no limitations/excellent health) and cover the dimensions of physical functioning, physical role, bodily pain, general health, vitality, social functioning, emotional role, and mental health. The SF-36 is the most widely used general health status instrument and has been translated into many languages [22]. The SF-36 allows for the scoring of the eight subscales mentioned above and the creation of two summary scales, the physical component summary (PCS) and mental component summary (MCS) scales [17].

#### 6-minute walk test.

The 6-Minute Walk Test (6MWT) has been included in a set of performance-based tests recommended by the OARSI for evaluating physical function in patients with KOA as a test of submaximal aerobic capacity and the ability to walk long distances [23]. Test will be performed indoors in accordance with the guidelines of the American Thoracic Society. Participants will walk at maximal effort, back and forth, in a 30-meter hallway turning round cones. Running will not be allowed, and assessors will provide standardized indications as “you’re doing well, keep it up” during the test at 1-minute intervals. The total distance walked in six minutes will be recorded [24].

#### 5-time sit-to-stand test.

5-time STS test is a reliable assessment tool and correlates with knee muscle strength, pain, stiffness, and physical function in patients with KOA [25]. During the test patients will be instructed to stand up five times from a straight-backed chair without using their arms and without bending their knees to 90°. They will cross their arms over their chest and aim to complete the task as quickly as possible by transitioning from a sitting position to a standing position.

#### OMERACT-OARSI responder criteria.

Patients showing a ≥ 50% reduction in VAS pain score and an absolute reduction of ≥ 20 points were defined as “absolute responders”; and patients showing a ≥ 20% reduction in VAS pain score and an absolute reduction of ≥ 10 points were defined as “responders” [26].

#### Analgesic usage.

All participants will be provided with a pain diary and will be asked to record their daily paracetamol usage in this diary.

#### The rate of adverse events.

The process of every adverse event (AE) will be recorded in detail, which includes its possible cause, therapeutical approaches, outcome and whether it is considered to be related to the intervention. Participants will also be monitored for AEs at each study visit after the enrollment. The study physician will assess the severity (i.e., severe, moderate, mild) and causality (i.e., definitely related, probably related, possibly related, unlikely related, not related) of the AEs and give advice accordingly.

### Data management

All data will be collected prospectively and stored in a secure, password-protected electronic database accessible only to authorized study personnel. Each participant will be assigned a unique study identification number to ensure anonymity. No personally identifiable information will be included in the analytic dataset.

Upon completion of the study and publication of the results, the fully anonymized dataset and corresponding metadata will be uploaded to a public open-access repository.

### Statistical analysis

All statistical analyses will be performed using the Statistical Package for the Social Sciences (SPSS) software, version 20.0 (IBM Corp., Armonk, NY, USA) and/or an equivalent validated statistical software package if required for model-based analyses. Descriptive data will be presented as means ± standard deviations or medians (interquartile ranges) depending on the distribution of the variable for continuous variables. Categorical variables will be described with frequency (percentage).The normality of continuous data will be tested using the Kolmogorov–Smirnov or Shapiro–Wilk tests.

The primary efficacy analysis will be based exclusively on the first treatment period (baseline to Month 3), prior to cross-over. The primary endpoint will be analyzed using a linear mixed-effects model (LMM), with WOMAC pain score as the dependent variable, treatment group and time as fixed effects, and participant as a random effect. Baseline WOMAC pain score and stratification factors (Kellgren–Lawrence grade and unilateral/bilateral disease status) will be included as covariates.

Longitudinal secondary analyses including post-cross-over assessments will be considered secondary and exploratory and will be performed using LMMs accounting for repeated measures within participants.

Missing data will be examined for extent and pattern. If missingness is limited, LMM analyses will be performed using all available observations under the missing-at-random assumption. If substantial missingness is observed, appropriate multiple-imputation methods will be considered as sensitivity analyses.

The primary analysis will follow the intention-to-treat (ITT) principle, including all randomized participants in the sequence to which they were originally assigned, regardless of protocol adherence, treatment completion, or cross-over participation. A per-protocol (PP) analysis will also be conducted as a secondary sensitivity analysis, including only participants who completed the first treatment period without major protocol deviations. The consistency between ITT and PP analyses will be examined to assess the robustness of treatment effects.

Sample size was determined a priori using G*Power 3.1.9.2 (Heinrich-Heine-Universität Düsseldorf, Germany) for the first-period parallel-group comparison. Assuming a standardized moderate effect size of 0.5 for the between-group difference in WOMAC pain response at Month 3, a two-sided alpha error of 0.05, and 80% power, 51 participants per group were required, resulting in a total sample size of 102 participants. This calculation was based on a standardized effect size rather than a specific WOMAC pain point difference or formally defined minimal clinically important difference.

## Discussion

This randomized, double-blind, placebo-controlled trial was designed to evaluate both the symptomatic effects and the safety profile of a single high-dose, cross-linked hyaluronic acid injection in knee osteoarthritis. The primary efficacy inference is based on the 3-month pre-cross-over comparison, while the subsequent cross-over period provides secondary/exploratory efficacy data and extended safety follow-up.

The study has several strengths: a placebo comparator, stratified randomization, comprehensive outcome assessment, and a one-year follow-up period. Importantly, the overall methodology aligns closely with the recommendations of the EUROVISCO Guidelines for the design and conduct of clinical trials assessing the disease-modifying effects of visco-supplementation, including the use of standardized outcome measures, appropriate patient selection, and rigorous safety monitoring. The inclusion of both patient-reported and performance-based assessments further strengthens the evaluation of treatment response.

Another notable strength of this protocol is the systematic monitoring of early and late adverse reactions. Given that the selected formulation contains a high concentration of hyaluronic acid and a greater degree of cross-linking, the study provides an opportunity to observe potential hypersensitivity or biocompatibility-related reactions that may not be detected with lower-dose preparations. Objective measurements such as dynamometric strength testing and timed functional tests add further robustness to the functional assessment, which is often underrepresented in visco-supplementation research.

However, certain limitations should be acknowledged. The 3-month interval between treatment periods may not fully eliminate residual effects from the initial injection. Although cross-over analyses and mixed-model approaches will be used to assess potential carry-over effects, complete washout cannot be guaranteed. Therefore, the primary efficacy analysis is restricted to the first treatment period before cross-over, whereas post-cross-over efficacy analyses will be considered exploratory. Another limitation is that the sample-size calculation was based on a standardized moderate effect size rather than a specific WOMAC pain point difference or formally defined minimal clinically important difference; therefore, the study may have limited power to detect smaller between-group differences. Additionally, the single-injection regimen may not directly reflect multi-injection protocols commonly used in clinical practice, and the single-center design may limit generalizability.

Despite these considerations, this study is expected to provide meaningful data on the long-term effectiveness and tolerability of high-dose cross-linked hyaluronic acid. The findings may help clarify the duration of clinical benefit after a single injection and offer safety information that can support clinical decision-making in the management of knee osteoarthritis.

## Supporting information

S1 File Medical device clinical research/studies application form. The original protocol approved by the ethics committee and regulatory authority before study initiation.
(DOCX)

S2 File Medical device clinical research/studies applicatıon form (English). The original protocol approved by the ethics committee and regulatory authority before study initiation (English).
(DOCX)

S3 File SPIRIT 2025 checklist. (DOCX)
