# Supplementary material for: Effectiveness and safety of intra-articular hyaluronic acid SEMICAL GEL-B CROSS therapy in knee osteoarthritis (SEM-ART1): Study protocol for a randomized, placebo controlled, double-blind, cross-over clinical trial
Source: PLoS One. 2026 Jul 9;21(7):e0353120. doi: 10.1371/journal.pone.0353120 (PMC13349147; doi:10.1371/journal.pone.0353120)
Supplement: S1 File — The original protocol approved by the ethics committee and regulatory authority before study initiation. (DOCX) [file pone.0353120.s001.docx]

| **Bölüm 1: Klinik Araştırma Tanımlaması** |
| --- |

**1.1.Sponsor/Destekleyici tanımlaması**

| Adı: | Semikal Teknoloji San. Ve Tic. A.Ş. |
| --- | --- |
| Açık Adresi: | Çünür Mah. 102. Cadde Teknokent 252/216 Merkez / Isparta |
| Telefon numarası: | 0 246 237 01 01 |
| E-posta: | [info@semical.com.tr](mailto:info@semical.com.tr) |

**Sponsorun irtibat kişisi**

| Adı Soyadı: | Aslıhan Kara |
| --- | --- |
| Telefon numarası: | 0532 749 57 98 |
| E-posta: | [aslihankara@semical.com.tr](mailto:aslihankara@semical.com.tr) |

**Sponsorun yasal temsilcisini tanımlaması**

| Yasal temsilciniz var mı?  Evet  Hayır |
| --- |
| Evet ise, yasal temsilci ile ilgili bilgileri doldurunuz (bölüm 1.2) |

**1.2. Yasal temsilci tanımlaması**

| Kuruluş adı: | Yasal temsilci yoktur. |
| --- | --- |
| Açık Adres: |  |
| Telefon numarası |  |
| E-posta |  |

**Yasal temsilcinin irtibat kişisi**

| Adı Soyadı: |  |
| --- | --- |
| Telefon numarası: |  |
| E-posta: |  |

**Klinik araştırma için irtibat kişisi**

| Sponsorun irtibat kişisiyle aynı |  |
| --- | --- |
| Yasal temsilcinin irtibat kişisiyle aynı |  |
| Diğer |  |
| “Diğer” i seçtiyseniz, lütfen bu klinik araştırma için diğer irtibat kişisi ile ilgili aşağıdaki bölümü doldurunuz. | |

**Klinik araştırma için diğer irtibat kişisi**

| Adı Soyadı: |
| --- |
| Açık Adresi: |

**1.3 Klinik araştırma tipi**

| CE işareti almamış klinik araştırma başvurusu |  |
| --- | --- |
| Piyasaya Arz Sonrası Çalışma (PMCF) başvurusu |  |
| Diğer klinik araştırma başvurusu |  |

**1.4 Başvuru Tipi**

| Avrupa Ekonomik Alanındaki (AEA) ilk başvuru |  |
| --- | --- |
| Ulusal düzeyde ilk başvuru (klinik araştırma AEA’ da halihazırda sunulmuş)  Bu durumda, lütfen verilen klinik araştırma kimliğini (CIV-ID) giriniz. |  |
| Yeniden başvuru  Bu durumda, mevcutsa lütfen CIV-ID’ yi giriniz. |  |

**1.5 AB/AEA/Birleşik Krallık (Kuzey İrlanda), Türkiye ve İsviçre dâhilindeki katılımcı ülkeler**

| Türkiye |
| --- |

**1.6 AB/AEA/Birleşik Krallık dışındaki katılımcı ülkeler**

| **Türkiye** |
| --- |

**1.7 Klinik araştırma planı (CIP)**

| CIP kodu: SEM-ART 1  CIP versiyonu: 1.0  CIP tarihi: 10.07.2023 |
| --- |

**1.8 Klinik araştırma başlığı**

| Tam başlık : SEMICAL JEL-B CROSS TEDAVİSİNİN DİZ OSTEOARTRİTİ OLAN HASTALARDA AĞRI, YAŞAM KALİTESİ VE KAS GÜCÜ ÜZERİNE ETKİSİNİN ve GÜVENLİLİĞİNİN DEĞERLENDİRİLDİĞİ TEK MERKEZLİ, ÇİFT KÖR, PLASEBO KONTROLLÜ ÇAPRAZ GEÇİŞLİ RANDOMİZE SATIŞ SONRASI KLİNİK TAKİP (PMCF) ÇALIŞMASI  Kısa başlık : SEMİCAL JEL-B CROSS TEDAVİSİNİN DİZ OSTEOARTRİTİNDE ETKİLİLİĞİ VE GÜVENLİLİĞİNİN DEĞERLENDİRİLDİĞİ SATIŞ SONRASI KLİNİK TAKİP ÇALIŞMASI    Meslekten olmayan kişilere yönelik başlık:  Diz kireçlenmesinde Semical Jel B Cross’un etkililiği ve güvenliliği |
| --- |

| **Bölüm 2: Klinik Araştırma Açıklaması** |
| --- |

**2.1 Bilimsel görüş**

| İmalatçı, Tıbbi Cihaz Yönetmeliğinin 61(2). Maddesinde belirtildiği gibi bir uzman heyete danışmış mı?  Evet  Hayır |
| --- |

**2.2 Klinik Araştırmanın Tasarımı**

| Temel klinik araştırma  Doğrulama araştırması  PMCF |
| --- |
| İnsan üzerinde ilk araştırma  İnsan üzerinde ilk araştırma değil |

**2.3 Tasarım metodolojisi**

| Vaka kontrol | | Kontrollü | Kesitsel | Çift kör |
| --- | --- | --- | --- | --- |
| Paralel | | Randomize | Açık |  |
| Diğer: |  | | | |

**2.4 Geliştirme evresi**

| Birinci evre  İkinci evre  PMCF |
| --- |

**2.5 Amaçlar ve Sonlanım noktaları**

| Birincil amaç/amaçlar: |
| --- |
| Birincil amaçlar: Bu çalışmanın amacı Semikal Teknoloji A.Ş. tarafından üretilip piyasaya sürülen SEMICAL GEL B-CROSS intraartiküler jellerin performans ve güvenliliğine ilişkin kısa ve uzun dönem klinik verilerin elde edilmesidir. |
| İkincil amaç/amaçlar: |
| Bilinen advers olay ve komplikasyonların takibi, önceden bilinmeyen advers olay ve komplikasyonların saptanması, gerçek hayat verilerine dayanarak yeni ortaya çıkan riskleri tanımlamak ve analiz etmek de amaçlanmaktadır |
| Diğer amaç/amaçlar: |
|  |
| Birincil sonlanım noktası/noktaları: |
| - Diz osteoartritinden kaynaklanan ağrının değişimi (Başlangıç, 3. Ay, 6. Ay, 9. Ay ve 12. Ayda WOMAC ağrı skorundaki değişim ile değerlendirilecektir |
| İkincil sonlanım noktası/noktaları: |
| - Alt ekstremite fonksiyonel skoru (WOMAC Fonksiyon indeksi ile başlangıç, 3. Ay, 6. Ay, 9. Ay ve 12. Ay) - Diz fonksiyonlarında düzelme (kas kuvvetinde, 5 kez otur-kalk testinde ve yürüme mesafesinde artış; Başlangıç, 3. Ay, 6. Ay, 9. Ay, 12. Ay ) - Analjezik tedavi ihtiyacında azalma (ağrı günlüğü ile) - Yaşam kalitesindeki değişim (SF-36 ile, Başlangıç, 3. Ay, 6. Ay,9. Ay ve 12. Ayda) - Hareket ve istirahat sırasındaki diz ağrısı değişimi (VAS ile, başlangıç, 3. Ay, 6, ay, 9. Ay, 12. Ay) - Enjeksiyon bölgesindeki lokal reaksiyonlar - Tüm çalışma periyodu boyunca advers olaylar |
| Diğer sonlanım noktası/noktaları: |
| Güvenlilik sonlanım noktaları:  Tedavinin tolere edilebilirliğinin ve güvenliliğinin advers olay ve komplikasyonların takibi ile değerlendirilmesi   - Erken dönem advers olay ve komplikasyonlar (enjeksiyon yerindeki lokal reaksiyonların sayısı ve şiddeti; 0. Gün – 10. Gün) - Geç dönem advers olay ve komplikasyonlar (0.gün – 12. Ay) |

**2.6 Klinik araştırmanın özeti**

| Genel özet:  SEM-ART 1 çalışması randomize kontrollü, çift kör, iki kollu ve tek merkezli bir çalışmadır. Azami 28 günlük bir tarama periyodu sonrasından Kellgren & Lawrence Evre II ve III diz osteoartritli hastalar 0. Gün olarak kabul edilen enjeksiyon gününde iki tedavi kolundan birine randomize olarak atanacaklardır. Bu tedavi kolları:   1. Grup: Tedavi Kolu – Çapraz bağlı intraartiküler hyaluronik asit (90 mg 3 ml çapraz bağlı hyaluronik asit) 2. Grup: Kontrol Kolu – Tedavi kolu ile aynı dozda intraartiküler izotonik salin çözeltisi (%0.9 sodyum klorür)   Çalışma tedavileri bir defa uygulanacaktır.  Tedavi enjeksiyonunun uygulanmasından 7-10 gün sonra bir güvenlik viziti yapılacak ve enjeksiyon yeri görsel olarak lokal reaksiyonlar bakımından değerlendirilecektir. Ayrıca, enjeksiyona bağlı advers olayların sorgulaması da yapılacaktır.  Hastalar; intraartiküler enjeksiyonun yapılmasından 3 ay sonra ilk kontrol ziyaretlerine geleceklerdir. Bu ziyarette, her iki gruptaki hastaların tedavi yanıtları önceden belirlenmiş objektif yanıt kriterleri ile çalışma tedavisine karşı kör olan ayrı bir değerlendirici tarafından değerlendirilecektir.  3.Ayda yapılan bu yanıt değerlendirmenin ardından her iki gruptaki hastaların aldıkları tedavi değiştirilerek diğer koldaki tedaviyi alacaklardır. Yani plasebo alanlar 3. Ayda hyaluronik asit enjeksiyonu olurken, hyaluronik asit enjeksiyonu yapılanlar da 3. ayda plasebo alacaklardır. Etkililik değerlendirmeleri üçer aylık aralarla yapılmaya devam edecektir.  Performans değerlendirmesi hastaların tedavi yanıtı ile gösterilecek olup 3. Ay, 6. Ay, 9. Ay ve 12. Ayda yapılacaktır. Bu değerlendirmelerde;   - WOMAC osteoartrit indeksi - Görsel Analog Skala ile son 1 aydaki ağrı düzeyinin değerlendirilmesi (aktivite ve istirahatteki ağrılar için 2 farklı değerlendirme alınacaktır). - Son 1 ayda analjezik ilaç kullanma ihtiyacının değerlendirilmesi (hasta günlüğü ile) - Yaşam kalitesi değerlendirilmesi (SF-36 anketi ile) - 6 dakika yürüme testi - 5 defa otur- kalk testi - Kas gücünün değerlendirilmesi (izometrik dinamometre/hand-held myometer ile)   Güvenlilik değerlendirmeleri çalışma boyunca her kontrolde advers olay ve yan etkilerin takibi, fizik muayene ve vital bulguların takibi ile yapılacaktır. Ayrıca intraartiküler enjeksiyonları takip eden ilk 48 saat içerisinde ciddi advers olayların yakın takibi yapılacak, enjeksiyona bağlı reaksiyonların değerlendirilmesi amacıyla tedaviden sonraki 7-10. Günde bir güvenlik takibi ziyareti gerçekleştirilecektir. |
| --- |
|  |

**2.7 Planlanan gönüllü sayısı**

| Avrupa’da :  Asya’da :  Afrika’da:  Kuzey Amerika’da:  Güney Amerika’da:  Okyanusya’da:  Türkiye’de: 102 |
| --- |
| Planlanan Toplam Gönüllü Sayısı: 102 |

**2.8 Klinik araştırmanın süresi**

| Tahmini Başlangıç Tarihi: 2023 yılı içerisinde başlanması hedeflenmektedir. En son tarih 31.12.2023 olarak belirlenmiştir.  Tahmini Bitiş Tarihi: 2024 yılında bitirilmesi hedeflenmektedir. |
| --- |

**2.9 Popülasyon**

**2.9.1 Tıbbi durum**

| Araştırılan durum herhangi bir hastalıkla ilgili mi?  Evet  Hayır |
| --- |
| Araştırılan durumlardan herhangi biri nadir bir hastalık mı?  Evet  Hayır |

**2.9.2 Tedavi alanı**

| Klinik araştırmanın kapsamına girdiği tedavi alanını (onkoloji, hematoloji vb.) belirtiniz: Fiziksel Tıp ve Rehabilitasyon, Ortopedi |
| --- |

**2.9.3 Gönüllülerin cinsiyeti**

| Kadın  Erkek |
| --- |

**2.9.4 Dâhil edilme kriterleri**

| - 18 yaş ve üzeri kadın ve erkek - American College of Rheumatology (ACR) kriterlerine göre klinik diz osteoartriti tanısı almış, antero-posterior diz grafisinde Kellgren & Lawrence evre II ve III osetoartrit bulguları olan - Tedavi öncesi ağrı derecelendirmesi VAS ile 4 puan ve üzerinde olan - İntraartiküler hyaluronik asit enjeksiyonu endikasyonu olan - Daha önce diz osteoartriti için konservatif tedavi görmiş ve yeterli yanıt elde edilememiş olan (konservatif tedavi; egzersiz, non-steroidal anti enflamatuar ilaç, fizik tedavi) - Vücut kitle endeksi 20-40 kg/m2 - Yazılı bilgilendirilmiş olur verebilen - Gebe veya emzirme döneminde olmayan - En az iki yıldır menopozda olan veya cerrahi olarak steril veya doğurganlık potansiyeli olup kabul edilebilir korunma yöntemlerini kullanmayı kabul eden, - Non-steroid anti-enflamatuar için arınma periyodu olan süreyi çalışma prosedürlerinin gerçekleştirilmesinden ve çalışma tedavisinin uygulanmasından önce tamamlamayı kabul eden hastalar (bu ifade, çalışma tedavisine yanıt değerlendirmelerinin yapılacağı ziyaretlerden en az 48 saat öncesine kadar herhangi bir NSAID ve başka ağrı kesici ajanları kullanmamak anlamına gelir). - Walker, baston, koltuk değneği vb yardımcı ekipman olmaksızın desteksiz kendi başına yürüyebilen, - Tedavi yanıtını değerlendirmek üzere kullanılan anket ve ölçekleri anlayabilmek ve doğru şekilde cevaplayabilmek için mental fonksiyonları yeterli olan (Mini Mental test skoru 24’ün üzerinde olan) - DN-4 anketi soruları ile ağrının nöropati kaynaklı olmadığı kanıtlanabilen, - Bilateral diz OA olanlar için; her iki diz VAS ağrı skorları benzer olmak koşuluyla (fark < 20) |
| --- |

**2.9.5 Hariç tutma kriterleri**

| - 18 yaşını doldurmamış bireyler, - Gebe, emziren ve 1 yıl içinde gebelik planı olan kadınlar, - Romatoid artrit, gut, psödogut, psöriazis, SLE, fibromiyalji gibi otoimmün veya inflamatuar romatizmal hastalık tanısı olan hastalar - Aktif inflamasyon veya enfeksiyonu olan hastalar, septik artritli hastalar - Antikoagülan tedavisi kullanan hastalar - Hyaluronik asit ve diğer yardımcı maddelere bilinen alerjisi olan hastalar - Tedavi etkililiğinin gözlenmesini engelleyebilecek diğer eklem hastalıkları - Çalışmaya dahil olmadan önceki 6 **ay** içinde diz içi intraartiküler steroid, PRP, kök hücre enjeksiyonu gibi diğer intraartiküler enjeksiyon tedavileri - Çalışma tedavisinin uygulanmasından **önceki 48 saat içinde aspirin**, asetaminofen veya başka bir non-steroid anti-enflamatuar ilaç kullanımı veya diğer opioid, kannabinoid ve pirazolon türevi analjeziklerin kullanımı - Çalışmaya dahil olmadan önceki **6 ay** içinde diz içi hyaluronik asit enjeksiyonu - Her iki dizden herhangi birine **son 1 yıl içinde açık cerrahi girişim** - Kalça ekleminde ileri derece (K&L Evre III- IV) osteoartrit olması - Hastanın bu çalışmaya dahil olmadan önceki 30 gün içinde başka bir çalışmada yer alması - Çalışma tedavisi ve prosedürleri ile çelişkili sonuçlar yaratabilecek başka tedavi veya uygulamalar (alternatif tıp, besin destekleri vb) - Periferal nöropati, vasküler yetmezlik, hemiparezi, sistemik kanama bozuklukları - Enjeksiyon yapılacak olan diz bölgesinde cilt hastalıkları veya enfeksiyon - Hastada sistemik hastalık, hemokromatoz veya diğer ağrılı kas iskelet hastalıklarına sekonder gelişen osteoartrit olması - Sudek atrofisi, Paget hastalığı gibi ağrılı kas iskelet hastalıkları, izole patellafemoral sendrom, kondromalazi - Hastanın, diz eklemindeki osteoartit semptomlarını değerlendirmesine engel olacak düzeyde ağrılı başka bir eklem artriti olması - Patellar şok testi pozitif olması veya enjeksiyon sorasında yapılan aspirasyonda >20 ml sinovial sıvı olması, - Alkol bağımlısı veya alkole bağlı karaciğer hastalığı olan hastalar - Ciddi kalp hastalığı, hepatik veya renal yetmezlik (AST, ALT ≥ x3 ULN; Serum Kreatinin >2 mg/dl - 10 dereceden yüksek varus yada valgus deformitesi veya 10 dereceden fazla eklem hareket kısıtlılığı bulunanlar - Günlük 5 mg prednizolondan daha yüksek dozda kortikosteroid veya başka immunsupresan ilaç tedavisi almakta olan hastalar - Bilişsel fonksiyonları çalışma sonuç ölçütlerinin değerlendirilmesi için yeterli olmayan hastalar (Mini Mental Stetement Score Test ile mental skoru 24’ten düşük hastalar) - Çalışma takiplerinin süreceği 12 ay boyunca planlanmış şehir veya ülke dışı kalıcı ikamet değişikliği, majör cerrahi operasyon, mahkûmiyet, askerlik, karantina vb durumu olmayan hastalar |
| --- |

**2.9.6 Klinik Araştırmaya Dahil Edilmesi Planlanan Gönüllü Grubu**

| Sağlıklı  Hastalar  Etkilenebilir özne  Kısıtlı gönüllüler  Çocuklar  Hamile kadınlar  Emziren kadınlar  Acil durumdaki hastalar  Diğer (lütfen belirtiniz) : |
| --- |

**2.9.7 Klinik araştırmaya dâhil edilmesi planlanan gönüllülerin yaş aralığı**

| İntrauterin Dönem  Yeni Doğan (0 ila 27 günlük)  Bebekler ve Küçük Çocuklar (28 günden 23 aya kadar)  Çocuklar (2 yaşından 11 yaşına kadar)  Ergenler (12 yaşından 17 yaşına kadar)  Yetişkinler (18 yaşından 84 yaşına kadar)  Yaşlılar (85 yaşından itibaren) |
| --- |

**2.10 Araştırma kapsamındaki cihazlar**

**2.10.1 Klinik araştırma kapsamında tıbbi cihaz ve in vitro tanı amaçlı tıbbi cihaz** **birlikte mi araştırılıyor?**

| Evet  Hayır  Evet ise, lütfen ilgili in vitro tanı amaçlı tıbbi cihazlar ile yürütülen performans değerlendirme çalışma numarasını belirtiniz: |
| --- |

**2.10.2 Klinik araştırma kapsamında tıbbi cihaz ve beşeri tıbbi ürün birlikte mi araştırılıyor?**

| Evet  Hayır  Evet ise, lütfen ilgili beşeri tıbbi ürün ile ilgili araştırma numarasını belirtiniz: |
| --- |

**2.11 Koordinatör/Sorumlu araştırmacı**

| Adı Soyadı: | Doç.Dr. Meral Bilgilisoy Filiz |
| --- | --- |
| Uzmanlık alanı: | Fiziksel Tıp ve Rehabilitasyon |
| Kurumu: | Antalya Eğitim ve Araştırma Hastanesi |
| Telefon numarası: | 0 505 647 58 40 |
| E-posta: | [mbilgilisoy@gmail.com](mailto:mbilgilisoy@gmail.com) |

| **Bölüm 3: Araştırma Cihaz/Cihazları** |
| --- |

**3.1 Araştırma amaçlı tıbbi cihaz**

**3.1.1 Cihazın kullanım amacı**

| Hastalarda, diz osteoartritinde ağrı tedavisi için endikedir. Hamile kadınlar ve çocuklarda hyaluronik asit kullanımı hakkında yeterli klinik veri bulunmadığından, bu hastalarda kullanılmamalıdır**.**  **Tam tanımlanmış tıbbi endikasyonlar:**  Dejeneratif veya travma sonrası hastalıklardan veya eklem hasarından kaynaklanan ağrılı eklem koşulları. Viskoelastik özelliği nedeniyle, bu ürün eklemlerin optimal reolojik ve fizyolojik durumlarını korumalarına yardımcı olur. Ürün sinovial sıvının karakterini arttırarak eklemleri korur ve eklem kıkırdağının onarımı için fizyolojik mekanizmaları uyarır. Bu özellik nedeniyle eklem fonksiyonunu arttırmaya ve ağrı semptomlarını azaltmaya yardımcı olur. |
| --- |

**3.1.2 Cihaz tipi**

| İmplante edilebilir | Sistem |
| --- | --- |
| Aktif cihaz | Tıbbi amaçlı olmayan (TCY Ek XVI) |
| Ölçüm fonksiyonu | Steril |
| Tekrar kullanılabilir cerrahi alet | Yazılım |
| Tıbbi ürünü tatbik etme veya uzaklaştırma amaçlı |  |

**3.1.3 Invaziv olma durumu**

| Bir invaziv tıbbi cihaz mı?  Evet  Hayır |
| --- |

**3.1.4 Cihaza ilişkin bilgiler**

| Jenerik adı (EMDN kodunun 4. seviyesine karşılık gelen cihaz grup adı):  A020107 – PREFILLED SYRINGES | | | | |
| --- | --- | --- | --- | --- |
|  | | | | |
| Cihazın ticari adı:  **Semical Jel B Cross** | |  | Modeli:  **3 ml/ 90 mg** |  |
| Cihaz adı: **İntraartiküler Hyaluronik Asit** |  | | | |
| Avrupa Tıbbi Cihaz Terminolojisi (EMDN):  **A02010799 – PREFILLED SYRINGES, OTHER** | | | | |
| Tıbbi cihazın sınıfı: **Sınıf III** | | | | |
|  | | | | |
| Sınıflandırma kuralı: **Kural 8** | | | | |
| Cihaz tanımı (Kullanılan malzeme dâhil olmak üzere klinik araştırma amaçlı cihazın ve bileşenlerinin genel açıklaması):  Semical Jel-B Cross İntraartiküler Jel, bakteriyel fermantasyonla elde edilen sodyum hyaluronat içerir ve intraartiküler bağ dokusunu desteklemek için viskoelastik jel olarak kullanılır. Steril ve apirojendir. Semical Jel-B Cross İntraartiküler Jel, fizyolojik tampon çözelti içinde çözünmüş 10-36 mg/ml sodyum hyaluronat (NaHA) içerir. Tüm çözelti, fermantatif orijinki sodyum hyaluronat ile düşük fosfatlı tampon çözelti içerir. | | | | |
| Cihazın kullanım amacı: Hastalarda diz osteoartritinde (OA) ağrı tedavisi için endikedir. Hamile kadınlar ve çocuklarda hyaluronik asit kullanımı hakkında klinik veri bulunmadığından, bu hastalarda kullanılmamalıdır. | | | | |
| Cihaz tıbbi madde/maddeler içeriyor mu?  Evet  Hayır  Evet ise, lütfen tıbbi madde/maddelerin adlarını giriniz: | | | | |
| Cihaz, bütünleşik bir parça olarak aşağıdakileri içermektedir veya bunlar kullanılarak imal edilmiştir:  Yardımcı işlevi olan cansız insan kaynaklı dokular veya bunların türevleri  Yardımcı işlevi olan cansız insan kaynaklı hücreler veya bunların türevleri  Yardımcı işlevi olan cansız hayvan kaynaklı dokular veya bunların türevleri  Yardımcı işlevi olan cansız hayvan kaynaklı hücreler veya bunların türevleri  Önceki noktalarda atıfta bulunulanlar hariç cansız biyolojik madde  Hiçbiri | | | | |
| Klinik araştırma amaçlı cihazın, Tıbbi Cihaz Yönetmeliği doğrultusunda alınmış CE işareti var mı?  Evet  Hayır  Evet ise, lütfen aşağıdaki bölüme gerekli bilgileri giriniz. | | | | |
| Sağlık Bakanlığı Ürün Takip Sistemine (ÜTS) kayıtlı mı?  Evet  Hayır  Evet ise, ürüne ait küresel ürün numarasını (barkod) lütfen belirtiniz ve ÜTS çıktısını başvuru dosyasına ekleyiniz.  Küresel Ürün No (Barkod): **8682190071187** | | | | |
| Cihazın klinik araştırma kapsamındaki kullanım amacı nedir?  CE işaretli cihaz, CE işareti kapsamı dışında kullanılacaktır.  CE işaretli cihaz, CE işareti kapsamında kullanılacaktır.  CE işaretli cihaz, CE işareti kapsamında kullanılacaktır ancak klinik araştırmada ek prosedürler uygulanacaktır. | | | | |
| Bu ek prosedürler ilave külfetli veya invaziv bir durum içeriyor mu?  Evet  Hayır  Evet ise açıklayınız:  Bu çalışmaya dahil olan hastalar, rutin klinik uygulamadan farklı olarak çalışmanın başında ve devamında üçer aylık aralıklarla aşağıdaki prosedürleri gerçekleştireceklerdir.   - Çalışma çapraz geçişli olarak tasarlandığından, her hastaya bir defa hyaluronik asit bir defa da placebo olmak üzere iki defa intraartiküler enjeksiyon uygulanacaktır. - 6 dakika yürüme testi - 5 defa oturup kalkma testi - Kas gücünün myometre ile ölçümü | | | | |
| Varsa, ilgili Onaylanmış Kuruluşla ilgili bilgiler:  Onaylanmış kuruluş numarası: **2292**  Onaylanmış kuruluş adı: **UDEM Uluslararası Belgelendirme A.Ş.** | | | | |

**3.2 Önceki klinik araştırmalar**

| Klinik araştırma amaçlı cihaz, daha önce bir klinik araştırmada kullanılmış mı?  Evet  Hayır  Evet ise, lütfen önceki klinik araştırmaların ilgili referans numaralarını yazınız, açıklayınız: |
| --- |

**3.3 Bilimsel görüş**

| Araştırma amaçlı cihaz için ulusal bir uzman heyetinden bilimsel görüş alındı mı?  Evet  Hayır |
| --- |

**3.4 Araştırma amaçlı cihazın imalatçısı**

| İmalatçı sponsor ile aynı mı?  Evet  Hayır  Hayır ise, lütfen bölüm 3.4.1 ve 3.4.2'de istenen bilgileri giriniz. |
| --- |

**3.4.1 İmalatçı bilgileri**

| Kuruluş adı: | Semikal Teknoloji San. Ve Tic. A.Ş. |
| --- | --- |
| Açık Adres: | Çünür Mah. 102. Cad. teknokent Sitesi No: 252/213 Merkez / Isparta |
| Telefon numarası | 0 246 237 01 01 |
| E-posta | [info@semical.com.tr](mailto:info@semical.com.tr) |

**İmalatçının irtibat kişisi**

| Adı Soyadı: | Aslıhan Kara |
| --- | --- |
| Telefon numarası: | 0532 749 57 98 |
| E-posta: | [Aslihan.kara@semical.com.tr](mailto:Aslihan.kara@semical.com.tr) |

**3.4.2 Varsa imalatçının yetkili temsilci**

| Kuruluş adı: |  |
| --- | --- |
| Açık Adres: |  |
| Telefon numarası |  |
| E-posta |  |

**Varsa imalatçının yetkili temsilcisinin irtibat kişisi**

| Adı Soyadı: |  |
| --- | --- |
| Telefon numarası: |  |
| E-posta: |  |

| Bu başvuru formunun ekine, bölüm 3 yinelenerek ilave cihazlar eklenebilir. |
| --- |

| **Bölüm 4: Karşılaştırma Durumu** |
| --- |

**4.1 Klinik araştırma kapsamında herhangi bir karşılaştırma var mı?**

| Evet  Hayır  Evet ise, lütfen 4.2 bölümüne istenilen bilgileri giriniz. |
| --- |

- 1. **Karşılaştırmanın türü**

| Başka bir tıbbi cihaz  Plasebo kontrollü  Tedavi yok  Tedavi yöntemi  Lütfen, seçilen türü açıklayınız: Plasebo olarak 3 ml fizyolojik serum çözeltisi kullanılacaktır. Plasebo, araştırma ürünü ile aynı görünümde, aynı şırıngalara doldurulmuş olacaktır. Ancak, hyaluronik asit ve fizyolojik serum çözeltisinin vizkoziteleri farklıdır ve bu fark enjeksiyonu yapan kişi tarafından fark edilebilir. Bu yüzden enjeksiyonlar tedavilere karşı kör olmayan bir yardımcı araştırmacı tarafından yapılacaktır. |
| --- |

**4.2.1 Karşılaştırma amaçlı kullanılan tıbbi cihazın durumu**

| Karşılaştırma amaçlı kullanılan tıbbi cihazın, Tıbbi Cihaz Yönetmeliği doğrultusunda alınmış CE işareti var mı?  Evet  Hayır  Evet ise, karşılaştırma amaçlı kullanılan tıbbi cihaz, imalatçısı tarafından belirlenen CE işareti kapsamında mı kullanılacak?  Evet  Hayır | | | | |
| --- | --- | --- | --- | --- |
| Jenerik adı (EMDN kodunun 4. seviyesine karşılık gelen cihaz grup adı): | | | | |
| Cihazın ticari adı: | |  | Modeli: |  |
| Cihaz adı: |  | | | |
| Avrupa Tıbbi Cihaz Terminolojisi (EMDN): | | | | |
| Tıbbi cihazın sınıfı: | | | | |
|  | | | | |
| Sınıflandırma kuralı: | | | | |
| Cihaz tanımı (Kullanılan malzeme dâhil olmak üzere klinik araştırma amaçlı cihazın ve bileşenlerinin genel açıklaması): | | | | |
| Cihazın kullanım amacı: | | | | |
| Cihaz tıbbi madde/maddeler içeriyor mu?  Evet  Hayır  Evet ise, lütfen tıbbi madde/maddelerin adlarını giriniz: | | | | |
| Cihaz, bütünleşik bir parça olarak aşağıdakileri içermektedir veya bunlar kullanılarak imal edilmiştir:  Yardımcı işlevi olan cansız insan kaynaklı dokular veya bunların türevleri  Yardımcı işlevi olan cansız insan kaynaklı hücreler veya bunların türevleri  Yardımcı işlevi olan cansız hayvan kaynaklı dokular veya bunların türevleri  Yardımcı işlevi olan cansız hayvan kaynaklı hücreler veya bunların türevleri  Önceki noktalarda atıfta bulunulanlar hariç cansız biyolojik madde  Hiçbiri | | | | |

| Bu başvuru formunun ekine, birden fazla karşılaştırma cihazı bulunması durumunda yeni bir bölüm 4 eklenmelidir. |
| --- |

| **Bölüm 5: Ulusal Bilgi** |
| --- |

**5.1. Araştırma merkezi bilgisi**

Lütfen klinik araştırmada yer alan tüm merkezlerin ve araştırmacıların bilgilerini giriniz.

| **Araştırma merkezinin adı** | **Adresi** | **Bu merkeze bağlı araştırmacılar** | **Araştırmacının araştırmadaki rolü** | **Araştırmacıların iletişim bilgileri** |
| --- | --- | --- | --- | --- |
| Antalya Eğitim ve Araştırma Hastanesi | Varlık Mah. Kazın Karabekir Cd. 07100 Antalya | Doç. Dr. Meral Bilgilisoy Filiz | Sorumlu Araştırmacı | Tel: +90 505 597 84 20  E-posta: [mbilgilisoy@gmail.com](mailto:mbilgilisoy@gmail.com) |
| Antalya Eğitim ve Araştırma Hastanesi | Varlık Mah. Kazın Karabekir Cd. 07100 Antalya | Dr. Öğr. Üyesi Hanife Hale Hekim | Yardımcı Araştırmacı | Tel:  E-posta: [hhhekim07@gmail.com](mailto:hhhekim07@gmail.com) |
| Antalya Eğitim ve Araştırma Hastanesi | Varlık Mah. Kazın Karabekir Cd. 07100 Antalya | Dr. Ahmet Bal | Yardımcı Araştırmacı | Tel: +90 542 102 48 07  E-posta: [balahmet93@gmail.com](mailto:balahmet93@gmail.com) |
|  |  |  |  |  |
|  |  |  |  |  |
|  |  |  |  |  |
|  |  |  |  |  |

**5.2 Etik kurul kararı bilgisi**

| Araştırmaya onay veren etik kurulun adı: **Antalya Eğitim Araştırma Hastanesi Klinik Araştırmalar Etik Kurulu**  Karar no:       Tarih: |
| --- |

**5.3 Sponsorun/Destekleyicinin statüsü**

| Ticari  Ticari değil |
| --- |

**5.4 AB üyesi ülke dâhilinde çalışmaya alınan gönüllülerin beklenen sayısı**

| Başvurunun yapıldığı AB üyesi ülkede çalışmaya kaç gönüllünün alınması bekleniyor?  **Sadece Türkiye’den 102 hasta dahil edilecektir.** |
| --- |

| **Bölüm 6: Başvuru Belgeleri** |
| --- |

**6.1 Bu bölümde yer alan ilgili tüm belgeler başvuru dosyasına eklenmelidir.**

| **İlgili Etik Kurul Kararı***  *Başvuru esnasında Etik kurul kararının aslı veya aslı gibidir onaylı örneği sunulmalıdır. Aslı gibidir onayı Etik Kurul Başkanı veya Etik Kurul Sekretaryası tarafından yapılmalıdır. Etik Kurul kararı içermeyen başvurular değerlendirmeye alınmayacaktır. |
| --- |
| **Araştırma Planı (CIP)***  Tarih:       Versiyon numarası:  *Asgari Tıbbi Cihaz Klinik Araştırma Planı (CIP) örneği doğrultusunda hazırlanmalıdır. Plan sponsor/destekleyici tarafından imzalanmalıdır. |
| **Bilgilendirilmiş Gönüllü Olur Formu (BGOF)***  Tarih:       Versiyon numarası:  *Asgari Bilgilendirilmiş Gönüllü Olur Formu örneğinin tüm maddelerini içerir şekilde hazırlanmalıdır. |
| **Olgu Rapor Formu (ORF)***  Tarih:       Versiyon numarası:  *“Araştırmadaki her bir gönüllüye ait verilerin ve diğer bilgilerin araştırma protokolünde tanımlandığı şekilde kaydının yapılması için hazırlanan basılı, optik veya elektronik belge” |
| **Araştırma Broşürü ***  Tarih:       Versiyon numarası:  *Asgari Tıbbi Cihaz Araştırmacı Broşürü örneği doğrultusunda hazırlanmalıdır. CE işareti taşımayan tıbbi cihaz ile yapılan klinik araştırmalar için başvuru dosyasına eklenmelidir. |
| **Klinik araştırma amaçlı cihaza ait etiket örneği***  ***** Tıbbi Cihaz Yönetmeliği doğrultusunda hazırlanmalıdır. CE işareti taşımayan tıbbi cihaz ile yapılan klinik araştırmalar için başvuru dosyasına eklemelidir. |
| **Klinik araştırma amaçlı tıbbi cihaza ait kullanım kılavuzu** |
| **Varsa karşılaştırma amaçlı tıbbi cihaza ait kullanım kılavuzu** |
| **Sigorta***  *CE işareti taşımayan veya kullanım kılavuzunda belirtilen amaç dışında kullanılacak tıbbi cihaz ile yapılan klinik araştırmalar için başvuru dosyasına eklenmelidir.  * Sigorta, [www.titck.gov.tr](http://www.titck.gov.tr) adresinde yer alan “Klinik Araştırmalarda Yapılacak Olan Sigorta Teminatına İlişkin Kılavuz” doğrultusunda hazırlanmalıdır. |
| **Araştırma Bütçesi***  *Bütçe formu, [www.titck.gov.tr](http://www.titck.gov.tr) adresinde yer alan güncel formatta yetkili kişiler (çok merkezli araştırmalar için koordinatör, tek merkezli araştırmalar için sorumlu araştırmacı ya da destekleyici*)* tarafından ıslak/e-imzalı olmalıdır. |
| **Destekleyiciye ait noter tasdikli imza sirküleri** |
| **Özgeçmiş formu***  *Çalışmada yer alan varsa koordinatör, sorumlu araştırmacı, yardımcı araştırmacı ve monitöre ait özgeçmiş formları başvuru dosyasına eklenmelidir.  **Özgeçmiş formu, [www.titck.gov.tr](http://www.titck.gov.tr) adresinde yer alan güncel formatta adı, soyadı ve unvanı el yazısı ile yazılmış, tarihli ve ıslak/e imzalı olmalıdır. |
| **Anabilim dalı başkanı veya eğitim sorumlusu tarafından onaylanan belge***  *Çalışmanın uzmanlık tezi veya akademik amaçlı olduğuna dair Anabilim Dalı Başkanı veya Eğitim Sorumlusu tarafından onaylanan ıslak imzalı belge sunulmalıdır. |
| **Varsa yetkilendirme belgeleri***  *Destekleyici tarafından herhangi bir yetkilendirme yapılmış ise ıslak/e-imzalı yetkilendirme belgelerini veya noter onaylı suretlerini dosyaya ekleyiniz. |
| **Varsa hasta kartı/günlüğü**  Tarih:       Versiyon numarası: |
| **Varsa gönüllü bilgilendirme metinleri, ilanlar, anketler** |
| **Türkiye İlaç ve Tıbbi Cihaz Kurumu internet sitesinde yayımlanan başvuru ücretinin yatırılarak karşılığında alınan dekont’un aslı ve bir örneği***  *Uzmanlık tezi veya akademik amaçlı başvurular hariç başvuru dosyasına eklenmeli ve ESY sistemine yüklenmelidir. |

İşbu başvuru ile birlikte ayrıntılı olarak sunduğum bilgi ve dokümanların doğru olduğunu ve talep edilen tüm bilgilerin sunulduğunu,

Araştırma amaçlı cihazın TS EN ISO 13485 Tıbbi Cihazlar - Kalite Yönetim Sistemleri ve kendisiyle ilgili diğer standartlara uygun olarak üretildiğini,

Gönüllülerin sağlığını ve güvenliliğini korumak için her türlü önlemin alınmış olduğunu,

Kişisel verilerin gizliliğinin korunması ile ilgili meri mevzuata riayet edileceğini taahhüt ederim.

Bu başvuru için toplanan tüm klinik araştırma bilgilerinin Avrupa Veri Koruma Mevzuatına (GDPR) uygun olarak yapıldığını onaylıyorum.

| **Başvuru Sahibi** |
| --- |
| Adı Soyadı: |
| Tarih (gün/ay/yıl olarak): |
| İmza (Islak /e-imzalı): |
