# Supplementary material for: Effectiveness and safety of intra-articular hyaluronic acid SEMICAL GEL-B CROSS therapy in knee osteoarthritis (SEM-ART1): Study protocol for a randomized, placebo controlled, double-blind, cross-over clinical trial
Source: PLoS One. 2026 Jul 9;21(7):e0353120. doi: 10.1371/journal.pone.0353120 (PMC13349147; doi:10.1371/journal.pone.0353120)
Supplement: S2 File — The original protocol approved by the ethics committee and regulatory authority before study initiation (English). (DOCX) [file pone.0353120.s002.docx]

| **Section 1: Clinical Research Definition** |
| --- |

**1.1. Sponsor/Supporting Organization Definition**

| Name: | Semikal Technology Industry and Trade Inc. |
| --- | --- |
| Full Address: | Çünür Mah. 102. Cadde Teknokent 252/216 Merkez / Isparta |
| Phone number: | 0 246 237 01 01 |
| Email: | [info@semical.com.tr](mailto:info@semical.com.tr) |

**Sponsor's contact person**

| Name: | Aslıhan Kara |
| --- | --- |
| Phone number: | 0532 749 57 98 |
| Email: | [aslihankara@semical.com.tr](mailto:aslihankara@semical.com.tr) |

**Identification of the sponsor's legal representative**

| Do you have a legal representative?  Yes No |
| --- |
| If yes, please fill in the information about the legal representative (section 1.2) |

**1.2. Legal representative identification**

| Organization name: | There is no legal representative. |
| --- | --- |
| Physical Address: |  |
| Phone number |  |
| Email |  |

**Legal representative's contact person**

| First Name Last Name: |  |
| --- | --- |
| Phone number: |  |
| Email: |  |

**Clinical trial contact person**

| Same as sponsor's contact person |  |
| --- | --- |
| Same as the legal representative's contact person |  |
| Other |  |
| If you selected "Other," please complete the following section regarding the other contact person for this clinical trial. | |

**Other contact person for the clinical trial**

| First Name Last Name: |
| --- |
| Street Address: |

**1.3 Clinical trial type**

| Clinical trial application without CE marking |  |
| --- | --- |
| Post-Market Surveillance (PMCF) application |  |
| Other clinical trial application |  |

**1.4 Application Type**

| First application in the European Economic Area (EEA) |  |
| --- | --- |
| First application at the national level (clinical trial already submitted in the EEA)  In this case, please enter the provided clinical trial identification number (CIV-ID). |  |
| Reapplication  In this case, please enter the CIV-ID if available. |  |

**1.5 Participating countries within the EU/EEA/United Kingdom (Northern Ireland), Turkey, and Switzerland**

| Turkey |
| --- |

**1.6 Participating countries outside the EU/EEA/United Kingdom**

| **Turkey** |
| --- |

**1.7 Clinical investigation plan (CIP)**

| CIP code: SEM-ART 1  CIP version: 1.0  CIP date: 10.07.2023 |
| --- |

**1.8 Clinical trial title**

| Full title: A Single-Center, DOUBLE-BLIND, PLACEBO-CONTROLLED CROSS-OVER RANDOMIZED POST-MARKET CLINICAL FOLLOW-UP (PMCF) STUDY  Short title: A POST-MARKET CLINICAL FOLLOW-UP STUDY EVALUATING THE EFFICACY AND SAFETY OF SEMİCAL GEL-B CROSS TREATMENT IN KNEE OSTEOARTHRITIS  Title for non-professionals:  The efficacy and safety of Semical Gel B Cross in knee osteoarthritis |
| --- |

| **Section 2: Clinical Study Description** |
| --- |

**2.1 Scientific opinion**

| Has the manufacturer consulted an expert committee as specified in Article 61(2) of the Medical Device Regulation?  Yes No |
| --- |

**2.2 Design of the Clinical Study**

| Basic clinical investigation Confirmatory investigation PMCF |
| --- |
| First study in humans Not the first study in humans |

**2.3 Design methodology**

| Case-control | | Controlled | Cross-sectional | Double-blind |
| --- | --- | --- | --- | --- |
| Parallel | | Randomized | Open |  |
| Other: |  | | | |

**2.4 Development phase**

| Phase 1 Phase 2 PMCF |
| --- |

**2.5 Objectives and Endpoints**

| Primary objective(s): |
| --- |
| Primary objectives: The objective of this study is to obtain short- and long-term clinical data on the performance and safety of SEMICAL GEL B-CROSS intra-articular gels manufactured and marketed by Semikal Technology Inc. |
| Secondary objective(s): |
| The study also aims to monitor known adverse events and complications, identify previously unknown adverse events and complications, and define and analyze emerging risks based on real-world data. |
| Other objective(s): |
|  |
| Primary endpoint(s): |
| - Change in pain due to knee osteoarthritis (will be assessed by change in WOMAC pain score at baseline, 3 months, 6 months, 9 months, and 12 months) |
| Secondary endpoint(s): |
| - Lower extremity functional score (using the WOMAC Function Index at baseline, 3 months, 6 months, 9 months, and 12 months) - Improvement in knee function (increase in muscle strength, 5 times sit-to-stand test, and walking distance; at Baseline, 3 Months, 6 Months, 9 Months, and 12 Months) - Decrease in analgesic treatment requirement (using pain diary) - Change in quality of life (using SF-36, at Baseline, 3 Months, 6 Months, 9 Months, and 12 Months) - Change in knee pain during movement and rest (using VAS, at Baseline, 3 Months, 6 Months, 9 Months, and 12 Months) - Local reactions at the injection site - Adverse events throughout the entire study period |
| Other endpoints: |
| Safety endpoints:  Evaluation of the tolerability and safety of the treatment by monitoring adverse events and complications   - Early adverse events and complications (number and severity of local reactions at the injection site; Day 0 – Day 10) - Late-phase adverse events and complications (Day 0 – 12 months) |

**2.6 Summary of the clinical study**

| General summary:  The SEM-ART 1 study is a randomized controlled, double-blind, two-arm, single-center study. Following a maximum 28-day screening period, patients with Kellgren & Lawrence Stage II and III knee osteoarthritis will be randomly assigned to one of two treatment arms on the day of injection, considered Day 0. These treatment arms are:   1. Group: Treatment Arm – Cross-linked intra-articular hyaluronic acid (90 mg 3 ml cross-linked hyaluronic acid) 2. Group: Control Arm – Intra-articular isotonic saline solution (0.9% sodium chloride) at the same dose as the treatment arm   Study treatments will be administered once.  A safety visit will be conducted 7-10 days after the treatment injection, and the injection site will be visually assessed for local reactions. In addition, adverse events related to the injection will be investigated.  Patients will attend their first follow-up visit 3 months after the intra-articular injection. At this visit, the treatment responses of patients in both groups will be assessed by a separate evaluator who is blinded to the study treatment, using pre-specified objective response criteria.  Following this response evaluation at 3 months, the treatment received by patients in both groups will be switched, and they will receive the treatment administered to the other arm. That is, those receiving placebo will receive hyaluronic acid injection at 3 months, while those receiving hyaluronic acid injection will receive placebo at 3 months. Efficacy evaluations will continue at three-month intervals.  Performance evaluation will be demonstrated by the patients' treatment response and will be conducted at 3 months, 6 months, 9 months, and 12 months. In these evaluations:   - WOMAC osteoarthritis index - Assessment of pain levels over the past month using the Visual Analog Scale (two separate assessments will be taken for pain during activity and at rest). - Evaluation of the need for analgesic medication in the last month (using a patient diary) - Quality of life assessment (using the SF-36 questionnaire) - 6-minute walk test - 5 sit-to-stand tests - Assessment of muscle strength (using an isometric dynamometer/hand-held myometer)   Safety assessments will be performed throughout the study at each visit by monitoring adverse events and side effects, physical examination, and vital signs. In addition, serious adverse events will be closely monitored within the first 48 hours following intra-articular injections, and a safety follow-up visit will be conducted on days 7-10 after treatment to evaluate injection-related reactions. |
| --- |
|  |

**2.7 Planned number of volunteers**

| Europe:  In Asia:  Africa:  North America:  South America:  In Oceania:  In Turkey: 102 |
| --- |
| Planned Total Number of Volunteers: 102 |

**2.8 Duration of the clinical trial**

| Estimated Start Date: It is planned to start within 2023. The latest date has been set as 12/31/2023.  Estimated End Date: The study is expected to be completed in 2024. |
| --- |

**2.9 Population**

**2.9.1 Medical Condition**

| Is the condition being studied related to any disease?  Yes No |
| --- |
| Is any of the conditions being studied a rare disease?  Yes No |

**2.9.2 Treatment area**

| Specify the treatment area covered by the clinical trial (oncology, hematology, etc.): Physical Medicine and Rehabilitation, Orthopedics |
| --- |

**2.9.3 Gender of Volunteers**

| Female Male |
| --- |

**2.9.4 Inclusion criteria**

| - Women and men aged 18 years and older - Diagnosed with clinical knee osteoarthritis according to the American College of Rheumatology (ACR) criteria, with Kellgren & Lawrence stage II and III osteoarthritis findings on anteroposterior knee radiography - Pre-treatment pain rating of 4 points or higher on the VAS - Indicated for intra-articular hyaluronic acid injection - Patients who have previously undergone conservative treatment for knee osteoarthritis and have not achieved an adequate response (conservative treatment: exercise, non-steroidal anti-inflammatory drugs, physical therapy) - Body mass index 20-40 kg/m2 - Able to provide written informed consent - Not pregnant or breastfeeding - Have been in menopause for at least two years or are surgically sterile or have fertility potential and agree to use acceptable methods of contraception - Patients who agree to complete the washout period for non-steroidal anti-inflammatory drugs prior to the completion of study procedures and administration of study treatment (this means not taking any NSAIDs or other pain relievers for at least 48 hours prior to visits where responses to study treatment will be evaluated). - Patients who can walk unaided without the use of assistive devices such as a walker, cane, crutch, etc., - Patients with sufficient mental function to understand and correctly answer the questionnaires and scales used to evaluate treatment response (Mini Mental Test score above 24), - Those for whom the DN-4 questionnaire questions confirm that the pain is not neuropathic in origin, - For those with bilateral knee OA: provided that the VAS pain scores for both knees are similar (difference < 20) |
| --- |

**2.9.5 Exclusion criteria**

| - Individuals under the age of 18, - Pregnant women, breastfeeding women, and women planning to become pregnant within 1 year, - Patients diagnosed with autoimmune or inflammatory rheumatic diseases such as rheumatoid arthritis, gout, pseudogout, psoriasis, SLE, fibromyalgia, - Patients with active inflammation or infection, including those with septic arthritis, - Patients receiving anticoagulant therapy, - Patients with known allergies to hyaluronic acid and other excipients - Patients with other joint diseases that may interfere with the observation of treatment efficacy - Other intra-articular injection treatments such as intra-articular steroids, PRP, or stem cell injections within 6 **months** prior to enrollment in the study - Use of **aspirin**, acetaminophen, or other non-steroidal anti-inflammatory drugs **within 48 hours prior to** the study treatment, or use of other opioid, cannabinoid, and pyrazolone derivative analgesics - Intra-articular hyaluronic acid injection within **6 months** prior to study enrollment - **Open surgical intervention** on either knee **within the past year** - Presence of advanced osteoarthritis (K&L Stage III-IV) in the hip joint - Participation in another study within 30 days prior to inclusion in this study - Other treatments or practices (alternative medicine, nutritional supplements, etc.) that could produce results conflicting with the study treatment and procedures - Peripheral neuropathy, vascular insufficiency, hemiparesis, systemic bleeding disorders - Skin diseases or infections in the knee area where the injection will be administered - The patient has osteoarthritis secondary to systemic disease, hemochromatosis, or other painful musculoskeletal diseases - Painful musculoskeletal diseases such as Sudeck's atrophy, Paget's disease, isolated patellofemoral syndrome, chondromalacia - The patient has another painful joint arthritis that prevents them from evaluating the symptoms of osteoarthritis in the knee joint - Positive patellar shock test or aspiration of >20 ml of synovial fluid during injection - Patients with alcohol dependence or alcohol-related liver disease - Severe heart disease, hepatic or renal insufficiency (AST, ALT ≥ x3 ULN; Serum Creatinine >2 mg/dl - Those with varus or valgus deformity greater than 10 degrees or joint motion restriction greater than 10 degrees - Patients receiving corticosteroid or other immunosuppressive drug therapy at a dose higher than 5 mg prednisolone daily - Patients whose cognitive functions are insufficient for the assessment of study outcome measures (patients with a Mini Mental State Examination score of less than 24) - Patients without planned permanent relocation outside the city or country, major surgical procedures, imprisonment, military service, quarantine, etc., for the 12-month duration of the study follow-ups |
| --- |

**2.9.6 Volunteer Group Planned for Inclusion in the Clinical Study**

| Healthy Patients Vulnerable subjects Restricted volunteers  Children Pregnant women Breastfeeding women Patients in emergency situations  **Other** (please specify): |
| --- |

**2.9.7 Age range of volunteers planned to be included in the clinical trial**

| Intrauterine Period  Newborn (0 to 27 days)  Infants and Young Children (28 days to 23 months)  Children (2 to 11 years old)  Adolescents (12 to 17 years old)  Adults (ages 18 to 84)  Elderly (85 years and older) |
| --- |

**2.10 Devices covered by the research**

**2.10.1 Are medical devices and in vitro diagnostic medical devices being studied together within the scope of the clinical research?**

| Yes No  If yes, please specify the performance evaluation study number conducted with the relevant in vitro diagnostic medical devices: |
| --- |

**2.10.2 Are medical devices and human medicinal products being investigated together within the scope of the clinical research?**

| Yes No  If yes, please specify the research number related to the human medicinal product: |
| --- |

**2.11 Coordinator/Principal Investigator**

| Name: | Assoc. Prof. Dr. Meral Bilgilisoy Filiz |
| --- | --- |
| Field of expertise: | Physical Medicine and Rehabilitation |
| Institution: | Antalya Training and Research Hospital |
| Phone number: | 0 505 647 58 40 |
| Email: | [mbilgilisoy@gmail.com](mailto:mbilgilisoy@gmail.com) |

| **Section 3: Research Device(s)** |
| --- |

**3.1 Medical device for research purposes**

**3.1.1 Purpose of the device**

| It is indicated for the treatment of pain in patients with knee osteoarthritis. As there is insufficient clinical data on the use of hyaluronic acid in pregnant women and children, it should not be used in these patients**.**  **Fully defined medical indications:**  Painful joint conditions resulting from degenerative or post-traumatic diseases or joint damage. Due to its viscoelastic properties, this product helps maintain the optimal rheological and physiological conditions of the joints. The product protects joints by enhancing the character of synovial fluid and stimulates physiological mechanisms for the repair of joint cartilage. Due to this property, it helps improve joint function and reduce pain symptoms. |
| --- |

**3.1.2 Device type**

| Implantable | System |
| --- | --- |
| Active device | Non-medical (TCY Annex XVI) |
| Measurement function | Sterile |
| Reusable surgical instrument | Software |
| For the purpose of applying or removing a medical product |  |

**3.1.3 Invasive status**

| Is it an invasive medical device?  Yes No |
| --- |

**3.1.4 Device information**

| Generic name (device group name corresponding to level 4 of the EMDN code):  A020107 – PREFILLED SYRINGES | | | | |
| --- | --- | --- | --- | --- |
|  | | | | |
| Commercial name of the device:  **Semical Gel B Cross** | |  | Model:  **3 ml/ 90 mg** |  |
| Device name: **Intra-articular Hyaluronic Acid** |  | | | |
| European Medical Device Nomenclature (EMDN):  **A02010799 – PREFILLED SYRINGES, OTHER** | | | | |
| Medical device class: **Class III** | | | | |
|  | | | | |
| Classification rule: **Rule 8** | | | | |
| Device description (General description of the device and its components, including the material used, for clinical research purposes):  Semical Gel-B Cross Intra-articular Gel contains sodium hyaluronate obtained by bacterial fermentation and is used as a viscoelastic gel to support intra-articular connective tissue. It is sterile and apyrogenic. Semical Gel-B Cross Intra-articular Gel contains 10-36 mg/ml sodium hyaluronate (NaHA) dissolved in a physiological buffer solution. The entire solution contains fermentative sodium hyaluronate and a low-phosphate buffer solution. | | | | |
| Intended use of the device: It is indicated for the treatment of pain in patients with knee osteoarthritis (OA). As there is no clinical data on the use of hyaluronic acid in pregnant women and children, it should not be used in these patients. | | | | |
| Does the device contain any medical substance(s)?  Yes No  If yes, please enter the names of the medicinal substance(s): | | | | |
| The device contains or is manufactured using the following as an integral part:  Inert human-derived tissues or their derivatives with an auxiliary function  Non-living human-derived cells or their derivatives with an auxiliary function  Non-living animal-derived tissues or their derivatives with an auxiliary function  Non-living animal-derived cells or their derivatives with an auxiliary function  Non-living biological material other than those referred to in the preceding points  None | | | | |
| Does the device intended for clinical research have a CE mark obtained in accordance with the Medical Device Regulation?  Yes No  If yes, please enter the required information in the section below. | | | | |
| Is it registered in the Ministry of Health Product Tracking System (ÜTS)?  Yes No  If yes, please specify the global product number (barcode) for the product and attach the ÜTS output to the application file.  Global Product Number (Barcode): **8682190071187** | | | | |
| What is the intended use of the device within the scope of the clinical trial?  The CE-marked device will be used outside the scope of the CE marking.  The CE-marked device will be used within the scope of the CE marking.  The CE-marked device will be used within the scope of the CE marking, but additional procedures will be applied in the clinical trial. | | | | |
| Do these additional procedures involve any extra burden or invasiveness?  Yes No  If yes, please explain:  Patients included in this study will undergo the following procedures at three-month intervals at the beginning and throughout the study, which differs from routine clinical practice.   - Since the study is designed as a crossover trial, each patient will receive two intra-articular injections: one with hyaluronic acid and one with placebo. - 6-minute walk test - 5 sit-to-stand tests - Measurement of muscle strength using a myometer | | | | |
| If applicable, information regarding the relevant Notified Body:  Approved Body number: **2292**  Name of the Notified Body: **UDEM International Certification Inc.** | | | | |

**3.2 Previous clinical studies**

| Has the device intended for clinical research been used in a previous clinical study?  Yes No  If yes, please list the relevant reference numbers of the previous clinical studies and explain: |
| --- |

**3.3 Scientific opinion**

| Has a scientific opinion been obtained from a national expert panel for the device intended for research purposes?  Yes No |
| --- |

**3.4 Manufacturer of the research device**

| Is the manufacturer the same as the sponsor?  Yes No  If no, please enter the information requested in sections 3.4.1 and 3.4.2. |
| --- |

**3.4.1 Manufacturer information**

| Company name: | Semikal Technology Industry and Trade Inc. |
| --- | --- |
| Full Address: | Çünür Mah. 102nd St. Teknokent Site No: 252/213 Merkez / Isparta |
| Phone number | 0 246 237 01 01 |
| Email | [info@semical.com.tr](mailto:info@semical.com.tr) |

**Manufacturer's contact person**

| Full Name: | Aslıhan Kara |
| --- | --- |
| Phone number: | 0532 749 57 98 |
| Email: | [Aslihan.kara@semical.com.tr](mailto:Aslihan.kara@semical.com.tr) |

**3.4.2 Authorized representative of the manufacturer, if any**

| Company name: |  |
| --- | --- |
| Physical Address: |  |
| Phone number |  |
| Email |  |

**If applicable, contact person of the manufacturer's authorized representative**

| First Name Last Name: |  |
| --- | --- |
| Phone number: |  |
| Email: |  |

| Additional devices can be added to this application form by repeating section 3. |
| --- |

| **Section 4: Comparison Status** |
| --- |

**4.1 Is there any comparison within the scope of the clinical study?**

| Yes No  If yes, please enter the required information in section 4.2. |
| --- |

- 1. **Type of comparison**

| Another medical device  Placebo-controlled  No treatment  Treatment method  Please explain the selected type: 3 ml of physiological saline solution will be used as a placebo. The placebo will be identical in appearance to the study product and will be filled into the same syringes. However, the viscosities of hyaluronic acid and physiological saline solution are different, and this difference may be noticeable to the person administering the injection. Therefore, injections will be performed by a non-blinded assistant researcher. |
| --- |

**4.2.1 Status of the medical device used for comparison**

| Does the medical device used for comparison have a CE mark obtained in accordance with the Medical Device Regulation?  Yes No  If yes, will the medical device used for comparison be used within the scope of the CE mark specified by the manufacturer?  Yes No | | | | |
| --- | --- | --- | --- | --- |
| Generic name (device group name corresponding to the 4th level of the EMDN code): | | | | |
| Commercial name **of** the device: | |  | Model: |  |
| Device name: |  | | | |
| European Medical Device Nomenclature (EMDN): | | | | |
| Medical device class: | | | | |
|  | | | | |
| Classification rule: | | | | |
| Device description (General description of the device and its components for clinical research purposes, including the materials used): | | | | |
| Intended use of the device: | | | | |
| Does the device contain any medicinal substance(s)?  Yes No  If yes, please enter the names of the medicinal substance(s): | | | | |
| The device contains or is manufactured using the following as an integral part:  Inert human-derived tissues or their derivatives with an auxiliary function  Non-living human-derived cells or their derivatives with an auxiliary function  Non-living animal-derived tissues or their derivatives with an auxiliary function  Non-living animal-derived cells or their derivatives with an auxiliary function  Non-living biological material, except those referred to in the preceding points  None | | | | |

| If there are multiple comparison devices, a new Section 4 should be added to the appendix of this application form. |
| --- |

| **Section 5: National Information** |
| --- |

**5.1. Research center information**

Please enter the information for all centers and researchers involved in the clinical research.

| **Name of the research center** | **Address** | **Researchers affiliated with this center** | **Researcher's role in the research** | **Researchers' contact information** |
| --- | --- | --- | --- | --- |
| Antalya Education and Research Hospital | Varlık Mah. Kazın Karabekir Cd. 07100 Antalya | Assoc. Prof. Dr. Meral Bilgilisoy Filiz | Principal Investigator | Tel: +90 505 597 84 20  Email: [mbilgilisoy@gmail.com](mailto:mbilgilisoy@gmail.com) |
| Antalya Training and Research Hospital | Varlık Mah. Kazın Karabekir Cd. 07100 Antalya | Dr. Hanife Hale Hekim, Assistant Professor | Research Assistant | Tel:  Email: [hhhekim07@gmail.com](mailto:hhhekim07@gmail.com) |
| Antalya Training and Research Hospital | Varlık Mah. Kazın Karabekir Cd. 07100 Antalya | Dr. Ahmet Bal | Assistant Researcher | Tel: +90 542 102 48 07  Email: [balahmet93@gmail.com](mailto:balahmet93@gmail.com) |
|  |  |  |  |  |
|  |  |  |  |  |
|  |  |  |  |  |
|  |  |  |  |  |

**5.2 Ethics committee decision information**

| Name of the ethics committee that approved the research: **Antalya Education and Research Hospital Clinical Research Ethics Committee**  Decision no: Date: |
| --- |

**5.3 Sponsor/Supporting Organization Status**

| Commercial Non-commercial |
| --- |

**5.4 Expected number of volunteers to be recruited within the EU member state**

| How many volunteers are expected to be recruited in the EU member state where the application is made?  **Only 102 patients from Turkey will be included.** |
| --- |

| **Section 6: Application Documents** |
| --- |

**6.1 All relevant documents listed in this section must be included in the application file.**

| **Relevant Ethics Committee Decision***  *The original Ethics Committee decision or a certified copy must be submitted during the application. The certification must be made by the Chair of the Ethics Committee or the Ethics Committee Secretariat. Applications without an Ethics Committee decision will not be considered. |
| --- |
| **Research Plan (CIP)***  Date: Version number:  *It should be prepared in accordance with the Minimum Medical Device Clinical Investigation Plan (CIP) example. The plan must be signed by the sponsor/supporting organization. |
| **Informed Consent Form (ICF)***  Date: Version number:  *It must be prepared to include all items of the Minimum Informed Consent Form example. |
| **Case Report Form (CRF)***  Date: Version number:  *"A printed, optical, or electronic document prepared to record the data and other information belonging to each volunteer in the study as defined in the study protocol." |
| **Research Brochure ***  Date: Version number:  *It should be prepared in accordance with the Minimum Medical Device Investigator Brochure example. It should be included in the application file for clinical trials conducted with medical devices that do not bear the CE mark. |
| **Sample label for the device intended for clinical research***  ***** Must be prepared in accordance with the Medical Device Regulation. Must be included in the application file for clinical trials conducted with medical devices that do not bear the CE mark. |
| **User manual for the medical device intended for clinical research** |
| **If applicable, user manual for the medical device used for comparison purposes** |
| **Insurance***  * Must be included in the application file for clinical trials conducted with medical devices that do not bear the CE mark or will be used for purposes other than those specified in the user manual.  * Insurance must be prepared in accordance with the "Guidelines on Insurance Coverage for Clinical Trials" available at [www.titck.gov.tr](http://www.titck.gov.tr). |
| **Research Budget***  *The budget form must be signed (wet/e-signature) by authorized persons (coordinator for multi-center studies, principal investigator or sponsor for single-center studies*)* in the current format available at [www.titck.gov.tr](http://www.titck.gov.tr). |
| **Notarized signature circular of the sponsor** |
| **Curriculum Vitae Form***  *CV forms for the coordinator, principal investigator, assistant researcher, and monitor, if any, involved in the study must be included in the application file.  **The CV form must be in the current format available at [www.titck.gov.tr](http://www.titck.gov.tr), with the name, surname, and title written in handwriting, dated, and wet/e-signed. |
| Document **approved by the department chair or education officer***  *A wet-signed document approved by the Department Chair or Education Officer stating that the work is a thesis or for academic purposes must be submitted. |
| **Authorization documents, if any***  *If any authorization has been granted by the sponsor, please attach the wet/e-signed authorization documents or notarized copies to the file. |
| **If available, patient card/journal**  Date: Version number: |
| **If available, voluntary information texts, announcements, surveys** |
| **The original and a copy* of the receipt obtained in exchange for the application fee published on the Turkish Medicines and Medical Devices Agency website**  *Except for specialist thesis or academic applications, it must be added to the application file and uploaded to the ESY system. |

I hereby declare that the information and documents I have submitted in detail with this application are correct and that all requested information has been provided,

The research device has been manufactured in accordance with TS EN ISO 13485 Medical Devices - Quality Management Systems and other relevant standards,

All necessary precautions have been taken to protect the health and safety of volunteers,

I undertake to comply with the applicable legislation regarding the protection of personal data privacy.

I confirm that all clinical research information collected for this application has been obtained in accordance with the European Data Protection Legislation (GDPR).

| **Applicant** |
| --- |
| Name and Surname: |
| Date (day/month/year): |
| Signature (Wet/e-signed): |
