# Supplementary material for: Effectiveness and safety of intra-articular hyaluronic acid SEMICAL GEL-B CROSS therapy in knee osteoarthritis (SEM-ART1): Study protocol for a randomized, placebo controlled, double-blind, cross-over clinical trial
Source: PLoS One. 2026 Jul 9;21(7):e0353120. doi: 10.1371/journal.pone.0353120 (PMC13349147; doi:10.1371/journal.pone.0353120)
Supplement: S3 File — (DOCX) [file pone.0353120.s003.docx]

# SPIRIT 2025 Checklist

Protocol Title: Effectiveness and safety of intra-articular hyaluronic acid SEMICAL GEL-B CROSS therapy in knee osteoarthritis (SEM-ART1): study protocol for a randomized, placebo controlled, double-blind, cross-over clinical trial

| Section/Item | Description | Addressed on page number |
| --- | --- | --- |
| 1. Title | Descriptive title with study design, population, intervention(s), and trial acronym if applicable | Page 1 |
| 2a. Trial registration | Trial identifier and registry name | Page 2 |
| 2b. WHO Trial Registration Dataset | All items required by WHO Trial Registration Data Set | Clinicaltrials.org  NCT06141018, https://clinicaltrials.gov/expert-search?term=NCT06141018 |
| 3. Protocol version | Version number and date | Page 2 |
| 4. Funding and support | Sources of funding, sponsor information, and role in protocol development | Page 2 |
| 5. Roles and responsibilities | Names, affiliations, roles of all protocol contributors | Page 20,21 |
| 6. Background and rationale | Scientific background and explanation of rationale | Page 6-7 |
| 7. Objectives and hypotheses | Specific aims and hypotheses | Page 7 |
| 8. Trial design | Type of trial, allocation ratio, masking, and framework | Page 2,8 |
| 9. Setting and locations | Settings and countries of recruitment and data collection | Page 2 |
| 10. Eligibility criteria | Inclusion and exclusion criteria | Page 8-11 |
| 11. Interventions | Detailed intervention for each arm to permit replication | Page 11-14 |
| 12. Outcomes | Primary, secondary, and exploratory outcomes with measurement timepoints | Page 14-18 |
| 13. Participant timeline | Schedule of enrolment, interventions, assessments | Page 12-13 |
| 14. Sample size | Estimated number of participants and justification | Page 19 |
| 15. Recruitment strategy | Planned recruitment methods | Page 10-12 |
| 16. Allocation sequence generation | Method of random sequence generation | Page10-11 |
| 17. Allocation concealment | Mechanism of allocation concealment | Page 10-11 |
| 18. Implementation | Who generates the sequence, enrolls, and assigns | Page 10-11 |
| 19. Blinding (masking) | Who is blinded, how, and under what circumstances unblinding is permitted | Page 10-11 |
| 20. Data collection methods | Plans for data collection and quality assurance | Page 12-13 |
| 21. Data management | Plans for data entry, coding, and storage | Page 2-3 |
| 22. Statistical methods | Methods for analysis of outcomes | Page 18-19 |
